# Use of a Low-Cost Portable 3D Virtual Reality Gesture-Mediated Simulator for Training and Learning Basic Psychomotor Skills in Minimally Invasive Surgery: Development and Content Validity Study

**DOI:** 10.2196/17491

**Published:** 2020-07-14

**Authors:** Fernando Alvarez-Lopez, Marcelo Fabián Maina, Francesc Saigí-Rubió

**Affiliations:** 1 Faculty of Health Sciences Universidad de Manizales Caldas Colombia; 2 Faculty of Psychology and Education Sciences Universitat Oberta de Catalunya Barcelona Spain; 3 Faculty of Health Sciences Universitat Oberta de Catalunya Barcelona Spain

**Keywords:** simulation training, minimally invasive surgery, user-computer interface, operating rooms, medical education, computer-assisted surgery

## Abstract

**Background:**

Simulation in virtual environments has become a new paradigm for surgeon training in minimally invasive surgery (MIS). However, this technology is expensive and difficult to access.

**Objective:**

This study aims first to describe the development of a new gesture-based simulator for learning skills in MIS and, second, to establish its fidelity to the criterion and sources of content-related validity evidence.

**Methods:**

For the development of the gesture-mediated simulator for MIS using virtual reality (SIMISGEST-VR), a design-based research (DBR) paradigm was adopted. For the second objective, 30 participants completed a questionnaire, with responses scored on a 5-point Likert scale. A literature review on the validity of the MIS training-VR (MIST-VR) was conducted. The study of fidelity to the criterion was rated using a 10-item questionnaire, while the sources of content-related validity evidence were assessed using 10 questions about the simulator training capacity and 6 questions about MIS tasks, and an iterative process of instrument pilot testing was performed.

**Results:**

A *good enough* prototype of a gesture-based simulator was developed with metrics and feedback for learning psychomotor skills in MIS. As per the survey conducted to assess the fidelity to the criterion, all 30 participants felt that most aspects of the simulator were adequately realistic and that it could be used as a tool for teaching basic psychomotor skills in laparoscopic surgery (Likert score: 4.07-4.73). The sources of content-related validity evidence showed that this study’s simulator is a reliable training tool and that the exercises enable learning of the basic psychomotor skills required in MIS (Likert score: 4.28-4.67).

**Conclusions:**

The development of gesture-based 3D virtual environments for training and learning basic psychomotor skills in MIS opens up a new approach to low-cost, portable simulation that allows ubiquitous learning and preoperative warm-up. Fidelity to the criterion was duly evaluated, which allowed a good enough prototype to be achieved. Content-related validity evidence for SIMISGEST-VR was also obtained.

## Introduction

### Background

The emergence of minimally invasive surgery (MIS) in the mid-1980s [1] led to an increase in the number of iatrogenic bile duct injuries, as many surgeons worldwide switched from the paradigm of open surgery to these procedures with no previous training [2,3]. In the wake of these developments, simulation became valuable as a tool for learning psychomotor skills in MIS and numerous studies have demonstrated its usefulness [4,5].

Simulators for skill learning in MIS can be classified into 3 large groups: (1) traditional box trainers, (2) augmented reality simulators (hybrids), and (3) virtual reality (VR) simulators [6,7]. The last two are expensive and are unavailable in most universities or hospitals in developing countries [8]. The first VR simulator for MIS training was MIS training–VR (MIST-VR) [9]. In 1998, evidence for the construct validity of the device was established [10]. Later, in 2002, the evidence for prediction validity was added [4,11]. Finally, from 2002 onward, the evidence for concurrent validity was also demonstrated [12,13]. Recent years have seen the development of low-cost, gesture-based touchless devices that can interact with 3D virtual environments, such as the Microsoft Kinect (MS Kinect, Microsoft Corp), Leap Motion Controller (LMC; Leap Motion Inc), and the Myo armband (Thalmic Labs, Kitchener) [14].

For the development of the simulator used in this study, the researchers adopted the design-based research (DBR) paradigm, also known as *design research*. DBR seeks the creation and validation of useful artifacts that do not exist in nature [15] and is described by Manson [16] as “a process of using knowledge to design and create useful artefacts, and then using various rigorous methods to analyze why, or why not, a particular artefact is effective. The understanding gained during the analysis phase feeds back into and builds the body of knowledge of the discipline.” DBR is a solution-oriented process that focuses on solving practical and complex real-world problems [17]. The artifacts created can be constructs (vocabulary and symbols), models (abstractions and representations), methods (algorithms and practices), *instantiations* (implemented and prototype systems), and better design theories [18,19]. To develop the simulator, the study followed the method proposed by Manson [16,20,21], in which using processes of abduction and deduction that detect errors in the design or function of the prototype, supports the development of improved versions until a sufficiently *good enough* functional product is obtained that can be subjected to validation studies [16,20-22]. These *good enough* devices are rarely complete and are functional systems ready to be used in practice; rather, they are innovations that define the ideas, practices, technical capabilities, and products using which systems analysis, design, implementation, and use are achieved effectively and efficiently [17].

### Objectives

The first aim of this study was to describe the development of a web-based 3D VR simulator mediated by a gesture interface device (LMC) for learning basic psychomotor skills in MIS, called gesture-mediated simulator for MIS–VR (SIMISGEST-VR). The device is characterized by its portability and low cost, as well as the possibility of learning and training at any time and place (ubiquitous learning). The second aim of this study was to evaluate fidelity to the criterion and to find sources of content-related validity evidence for SIMISGEST-VR.

## Methods

### Overview

This is a descriptive report of the development, using a DBR paradigm, of a gesture-mediated simulator for learning basic psychomotor skills and of the prospective evaluation of the data obtained from Likert scale surveys to evaluate fidelity to the criterion and the sources of content-related evidence. To this end, the study participants rated fidelity to the criterion using a 10-item questionnaire about its ease of use, relevance as a tool for simulation in MIS, degree of correspondence between the movements of the forceps and their representation in the virtual space, and feedback. The sources of content-related validity evidence were (1) a literature review on a previously validated tool, the MIST-VR, and (2) an expert panel that answered 10 questions about the training capacity and 6 questions about each proposed task, with responses scored on a 5-point Likert scale that rated the extent to which the test content represented the domain evaluated. An iterative process of simulator development was performed using pilot testing by surgeons, engineers, and education experts until a *good enough* prototype was achieved.

The hypotheses were as follows:

- It is possible to develop a portable, low-cost, gesture-mediated simulator using the LMC for training and learning basic psychomotor skills in MIS.
- The 3D virtual environment and the proposed tasks showed fidelity to the criterion.
- It is possible to demonstrate sources of evidence for the content validity of the test items.

The first step of the validation process was to define the construct and proposed interpretation. In this study, the general construct is psychomotor skills in surgery, specifically basic psychomotor skills in MIS. The assumptions and proposed interpretations are that the 3D virtual environment is faithful to the criterion and the tasks adapted from the MIST-VR represent the construct that is intended to be measured. The instrument under investigation is a contactless, gesture-mediated simulator that uses the LMC (construct context). To determine the current use of gesture-mediated interfaces in surgery, especially in the field of surgical simulation, a systematic literature review was conducted [14]. Finally, as content-related validity evidence was collected, the goal was to identify whether there were any areas of construct underrepresentation or construct irrelevance.

### Phase 1: Initial Development of the Gesture-Mediated Simulator for Minimally Invasive Surgery-Virtual Reality

To develop a new type of web-based 3D VR simulator mediated by a gesture interface device (LMC) for learning basic psychomotor skills in MIS, a group consisting of a pediatric surgeon, systems engineer, industrial designer, and specialists in education was formed. The following technical elements were assembled: an electronic device (LMC), a computer program for the development of the 3D environment, a computer, hardware devices with no electronic components, and a database administrator.

#### Electronic Device: Leap Motion Controller

In May 2012, a sensor was launched based on the principle of infrared optical tracking, which detects the positions of fine objects such as fingertips or pen tips in a Cartesian plane. Its interaction zone is an inverted cone of approximately 0.23 m³, and it has a motion detection range that fluctuates between 20 mm and 600 mm [23,24]. This sensor measures 76 mm × 30 mm × 13 mm and weighs 45 g. It has 3 infrared emitters and 2 infrared cameras that capture the movements generated within the interaction zone [25,26]. The manufacturer reports an accuracy of 0.01 mm for fingertip detection, although one independent study showed an accuracy of 0.7 mm [27]. Although the LMC is designed mainly to detect the motion of the hands, it can track objects such as pencils and laparoscopic surgical forceps [28,29].

The LMC has been used as a tool to manipulate medical images in the fields of interventional radiology and image-guided surgery or when there is a risk of contamination through contact (eg, autopsy rooms). It has also been used for touchless control of operating lights and tables and simulation in MIS and robotic surgery using physical or VR simulators [14,28].

#### Unity3D and Development of the Web-Based Virtual Environment Based on Minimally Invasive Surgery Training–Virtual Reality Tasks

The 3D virtual environment with MIS tasks was created using a tool for developing games, Unity3D, which allows apps to be developed that are independent of the operating system or device [30].

The basis for the development of this environment was the MIST-VR, presented in 1997. This device is a low-cost, nonprocedural simulator that provides a large variety of metric data for analysis [31] and generates simple and abstract images that allow the training and learning of basic psychomotor maneuvers that cross many surgical disciplines [9,32,33]. The simple images allow novice learners to progress rapidly in the early phase of the basic psychomotor skills learning curve [34-36], although detailed performance analysis and feedback allow them to train alone, with no need for specialized instructors [37].

The basic psychomotor skills in MIS that can be learned using the MIST-VR are navigation-coordination, touching, grasping, stretching-traction, translocation, and electrocautery [38].

#### Computer

The computer displays the 3D virtual environment, records the metrics, stores them on a database, and provides feedback using graphs that show the score obtained after each exercise. The virtual environment developed runs on both PC and iOS operating systems.

#### Hardware Devices

The mechanical devices are represented by 2 MIS forceps that do not need to be functional, 2 support devices for the forceps with an entry trocar simulator, 1 support device for the LMC, and 1 pad for mounting the support devices.

During the development of the virtual environment, the types of specificity recommended by Bowman et al [39] were applied:

- *Application:* To design a web-based 3D virtual environment for basic psychomotor skills training in MIS
- *Domain:* Basic psychomotor skills in MIS
- *Tasks:* 6 tasks described in the MIST-VR were adopted
- *Device:* LMC, LEAP
- *Users:* Surgeons in training for learning basic psychomotor skills in MIS

### Phase 2: Evaluation of Fidelity to Criterion, Content-Related Validity Evidence

#### Subjects

The study was performed over a period of 3 months at different locations: XXXIV Brazilian Congress of Paediatric Surgery (Campo Grande, Brazil); Hospital Vall d’Hebron (Barcelona, Spain); and Hospital Infantil de la Cruz Roja (Manizales, Colombia). A total of 22 experienced surgeons (performed more than 100 MIS procedures) and 8 pediatric and general surgery residents (referent group, performed less than 100 MIS procedures) assisted in an informative session on the characteristics of the project, watched a demonstration video of the different tasks supported by the simulator, and had 2 opportunities to perform each of the tasks on the simulator. The performance metrics were not taken into account during this study, as the emphasis was placed on the assessment of the tool by those surveyed.

#### Content-Related Validity Evidence for a Previously Validated Tool

The first source of content validity for the SIMISGEST-VR sought to identify the main sources of validity evidence for the MIST-VR, as well as the studies that have demonstrated such validity.

#### Questionnaire

First, a demographic survey was administered that included questions on the level of training as a surgeon and level of experience in MIS, as well as experience with video games. The different factors in the evaluation of fidelity to the criterion and content validity study were assessed using a Likert scale, where 1=strongly disagree, 2=disagree, 3=neither agree nor disagree, 4=agree, and 5=strongly agree [40].

The questionnaire to assess fidelity to the criterion evaluated 10 aspects, while the content validity rated the training capacity and the tasks. In terms of the training capacity, 6 aspects were evaluated, and each of the 6 tasks (Table 1) was assessed based on whether or not it represented a specific surgical maneuver (Multimedia Appendix 1).

#### Simulator, Hardware, and Software

This study used SIMISGEST-VR with 6 tasks and their respective metrics and feedback. The hardware and software components of the simulator are described in phase 1: Development of SIMISGEST-VR of this paper.

#### Statistics

Normality was tested using the Shapiro-Wilk test. The distribution of the variables was not normal. The Likert scale median and interquartile range differences between the levels of education and experience were compared using the Kruskal-Wallis test. A statistically significant level <0.05 was established. The analysis was performed using Stata version 15.0 (StataCorp).

## Results

### Phase 1: Development of Gesture-Mediated Simulator for Minimally Invasive Surgery–Virtual Reality

#### The Virtual Environment

**Figure 1:**
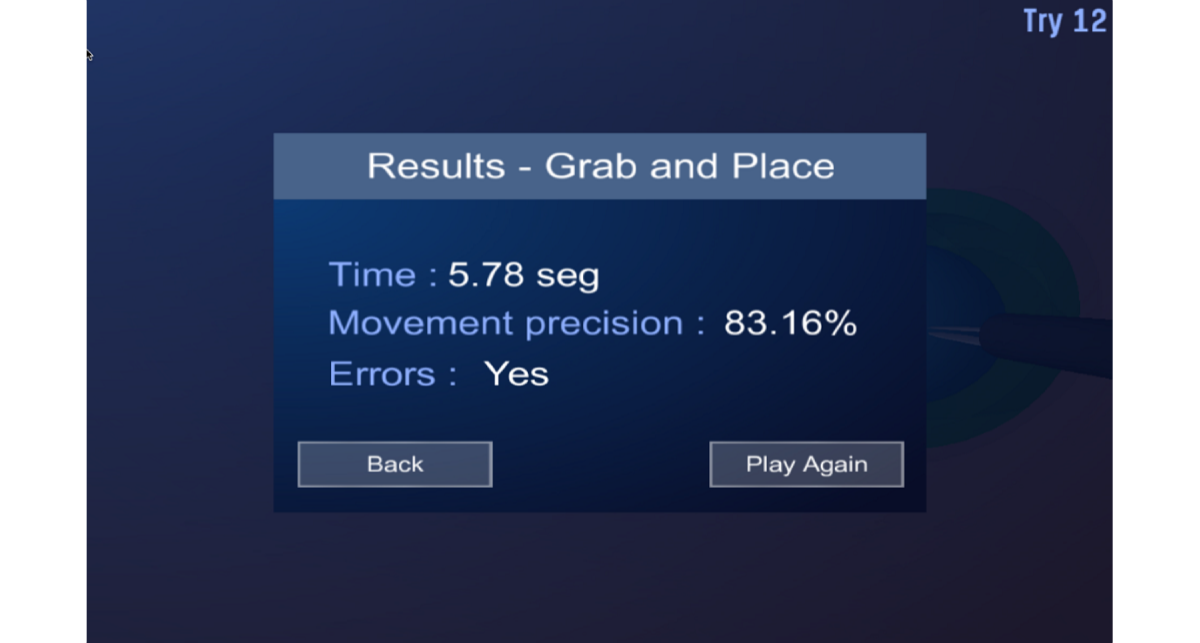
Immediate feedback.

**Figure 2:**
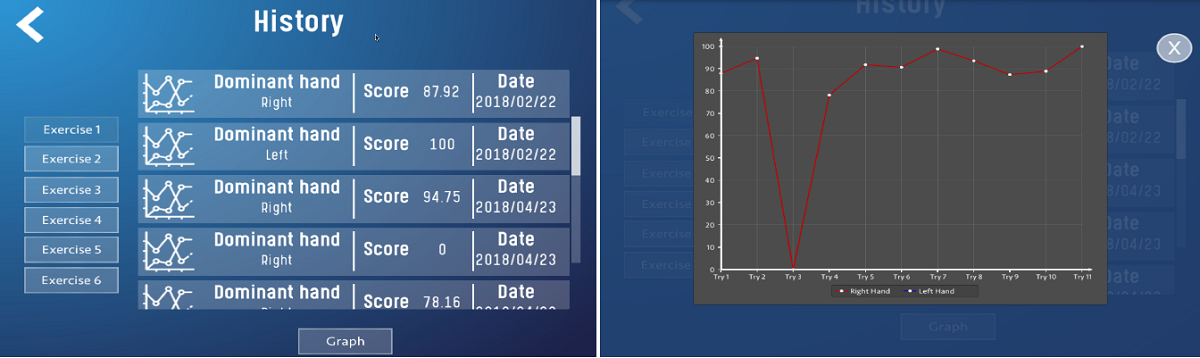
Performance history and terminal feedback curve.

The virtual environment consists of the following modules:

- *Registration:* Collects the user’s demographic information and stores it in the database
- *Tutorial:* Presents demonstration videos of the exercises
- *Test (tasks):* Supports 6 tasks, each of which corresponds to a surgical equivalent (Table 1) [9,41]
- *Performance graphs:* When an exercise is completed, the platform displays the results of the metrics in terms of the time taken to perform the exercise, precision of movement, and presence or absence of errors (immediate feedback; Figure 1). In this module, the student can look up the score obtained after each exercise and check whether or not their performance has improved (terminal feedback; Figure 2).

Except for Task 3, all tasks have the option of configuring the dominant hand during the exercise. Task 3 requires the simultaneous use of both hands and therefore both play a dominant function.

The web-based virtual environment runs on PC and iOS platforms.

These exercises are based on the instructional strategy known as *drill and practice*, which promotes the acquisition of knowledge or skill through repetitive practice [42].

**Table 1:** Description of the tasks and their surgical equivalents.

Task^a^	Description	Surgical equivalent
Task 1: Grip and placement	Take the sphere with one hand and move it to a new location within the workspace	Gripping and retraction of a tissue to a given position, placement of clips and hemostasis, and use of extractor bags
Task 2: Transfer and placement of an object	Take the sphere, transfer it to another instrument, and place it inside a hollow cylinder	Transfer of a needle between a clamp and a needle holder
Task 3: Cross	Instruments travel along a surface in a 3D cylinder	Small intestine exploration
Task 4: Removal and reinsertion of instruments	Removal of the instruments from the operative site and reinsertion	One instrument stabilizes one organ while the other is removed from the field and reintroduced
Task 5: Diathermy	Cauterize a series of targets located in a fixed sphere	Cauterize a bleeding blood vessel
Task 6: Target manipulation and diathermy	Take the sphere with the instrument and place it inside a virtual space represented by a cube. Cauterize a series of targets with the other hand	Present and set a target to cauterize

^a^Adapted from [9].

#### Metrics

The metrics were established using 5 parameters:

1. *Time:* Time taken from starting the task until completion. The time is measured only for each individual task.
2. *Efficiency of movement for the right and left hand:* This is the time during which the tip of the forceps is outside the ideal path, that is, the difference between the actual and the ideal path length [10,43].
3. *Economy of diathermy:* If contact occurs with the target for more than 2 sec during the diathermy, it is considered excess burn time and is penalized as a specific error.
4. *Error:* The following were defined as errors [43,44]: contact of the target with a part of the forceps other than the tip: all exercises; contact of the instrument with the limits of the virtual working space: all exercises; number of contacts of the instrument with the target sphere: exercises 1 and 2; number of contacts of the instrument or the sphere with the container margins: exercises 1 and 2; number of times that the instruments made undue contact between them: exercises 3 and 4; number of times that the instruments exceeded the number of contacts permitted with the oval: exercise 4; time during which the tip of the instrument remained outside the ideal path for the exercise: exercises 1, 2, 4, and 6; diathermy of the sphere outside the stated objectives: exercises 5 and 6; excess burn time: exercises 5 and 6.
5. *Final score:* The final score is obtained from the sum of the results of the efficiency of movement for each hand plus the economy of diathermy and from the errors generated in each exercise. Each exercise generates different types of errors, and each error is assigned a value between 1 and 5, where 1 is the least important and 5 is the most important. For example, diathermy outside the assigned targets means an error with an assigned score of 5. The number of errors committed in each of the exercises is then counted, and this figure is multiplied by the value assigned to each error. Finally, all the figures obtained in each of the errors are added, and the final score results from subtracting the sum obtained from 100. This is expressed in the following formula: 100–∑(error×value). Thus, a higher score indicates better performance.

#### Feedback

The haptic sensation and the concurrent feedback are simulated using sound signals, color changes in the objects, and movement of the object when an undue collision occurs between the different components of the environment or when an error occurs during the exercise. At the end of each task, the system provides information on the presence or absence of errors, the efficacy and efficiency, and the time required (immediate feedback). At the end of each training session, the system provides a series of graphs and tables that show the performance over time; this is the terminal feedback (Figures 1 and 2).

#### SQLite Database Engine

The data generated by the program were initially stored on an independent Structured Query Language database engine. However, during the development, this database was integrated into the virtual environment, which facilitated the acquisition of the users’ demographic data, registration of all the data provided by the metrics, and generation of reports of the users’ demographic and performance data. This information is stored on the computer on which the tests are performed.

#### Hardware

Two laparoscopic forceps were used. These MIS forceps did not need to be functional.

**Figure 3:**
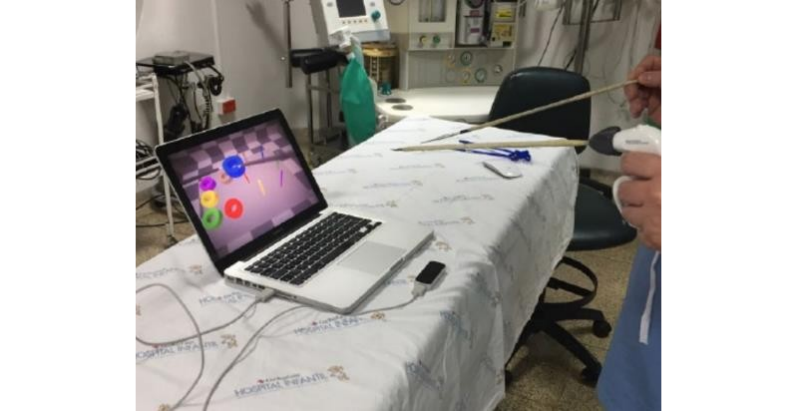
Initial version of the prototype without support devices for the forceps.

**Figure 4:**
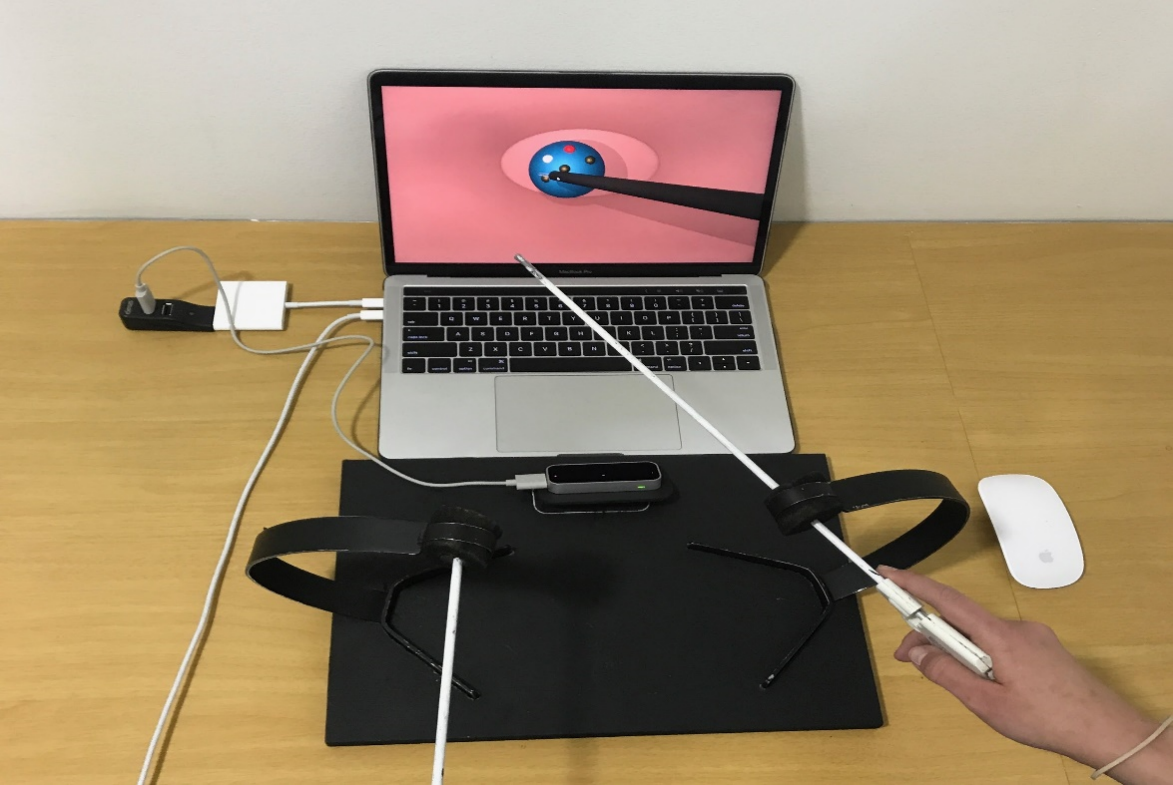
The final version of the simulator once the nonelectronic hardware devices had been added: the pad and support devices for the forceps and the Leap Motion Controller.

In the initial phase of development, the researchers used a prototype that did not have support devices (Figure 3), but it soon became evident that the fulcrum effect was not being reproduced. For this reason, they designed support devices for the forceps, which simulate the entry portal to the abdomen (Figure 4). These devices, while generating friction when inserting and removing the forceps, limit the moment of the arms, as occurs in real surgical procedures. During the process of designing these devices, principles were prioritized, such as noninterference with the forceps reading by the LMC, portability, and low cost. A pad for mounting the support devices and the LMC was also designed, which had a 45-degree tilt on a horizontal plane.

**Figure 5:**
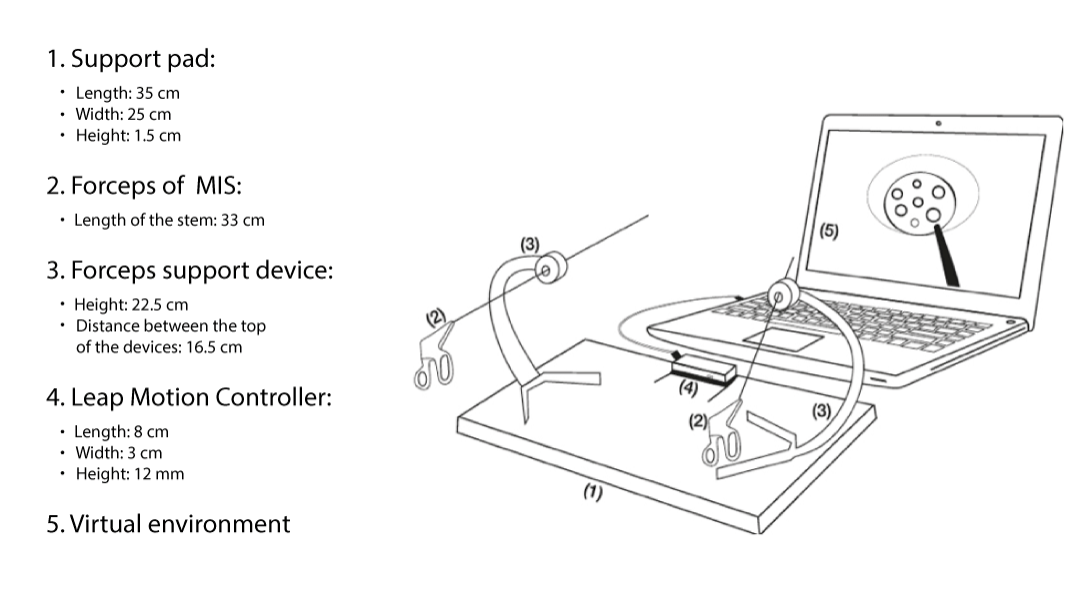
Diagram of the artefact.

The final artifact with all its components assembled is shown in Figure 5. It shows the fixing pad (1) for the LMC and the mounting support devices (3) for the MIS laparoscopic forceps (2), which allow simulation of the fulcrum effect; the LMC (4), responsible for detecting the movements of the instruments; and the computer, which using the software programs administers the virtual environment and the metrics and provides feedback and the final performance score on the screen (5), where the 3D virtual environment is displayed.

In Figure 4, the LMC has a 45-degree tilt toward the screen with respect to the horizontal plane. This arrangement was the result of a process of trial and error, which showed that setting the LMC at this angle with respect to the horizontal plane ostensibly improved the detection of the forceps. Another significant change during the design was that the original black color of the shaft of the forceps did not facilitate reading by the LMC [45]; therefore, they were painted white in the final prototype (Figure 4).

**Figure 6:**
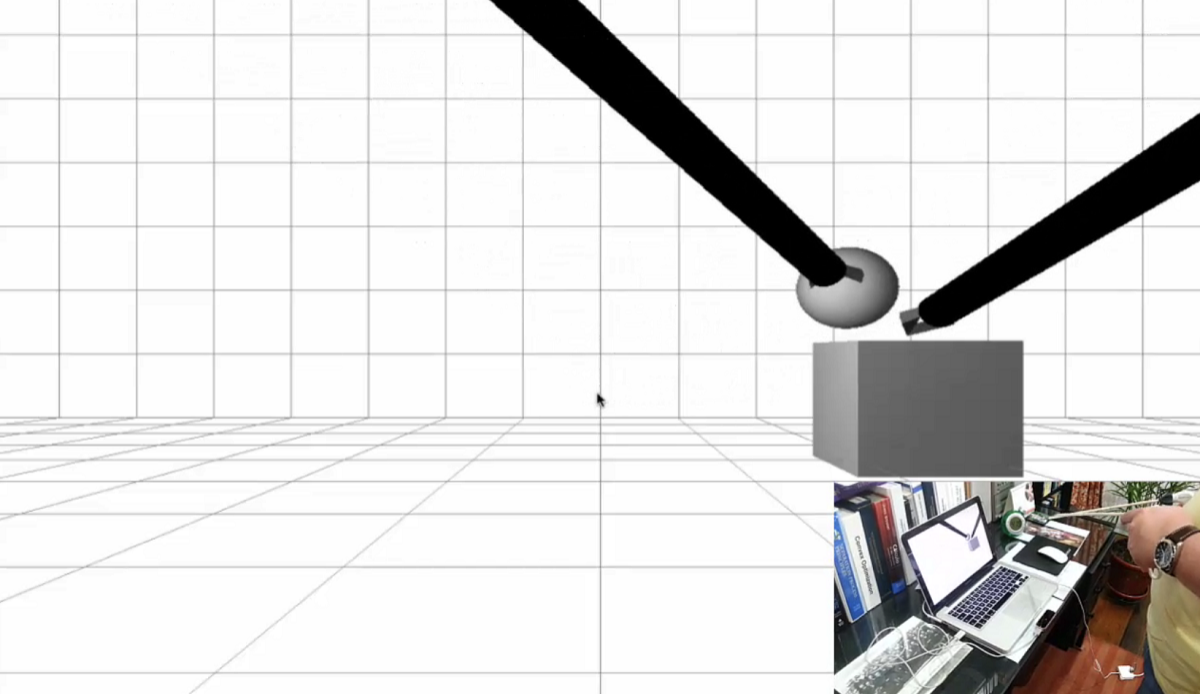
Initial attempts at interaction between minimally invasive surgery forceps and Leap Motion Controller within a basic 3D virtual environment.

**Figure 7:**
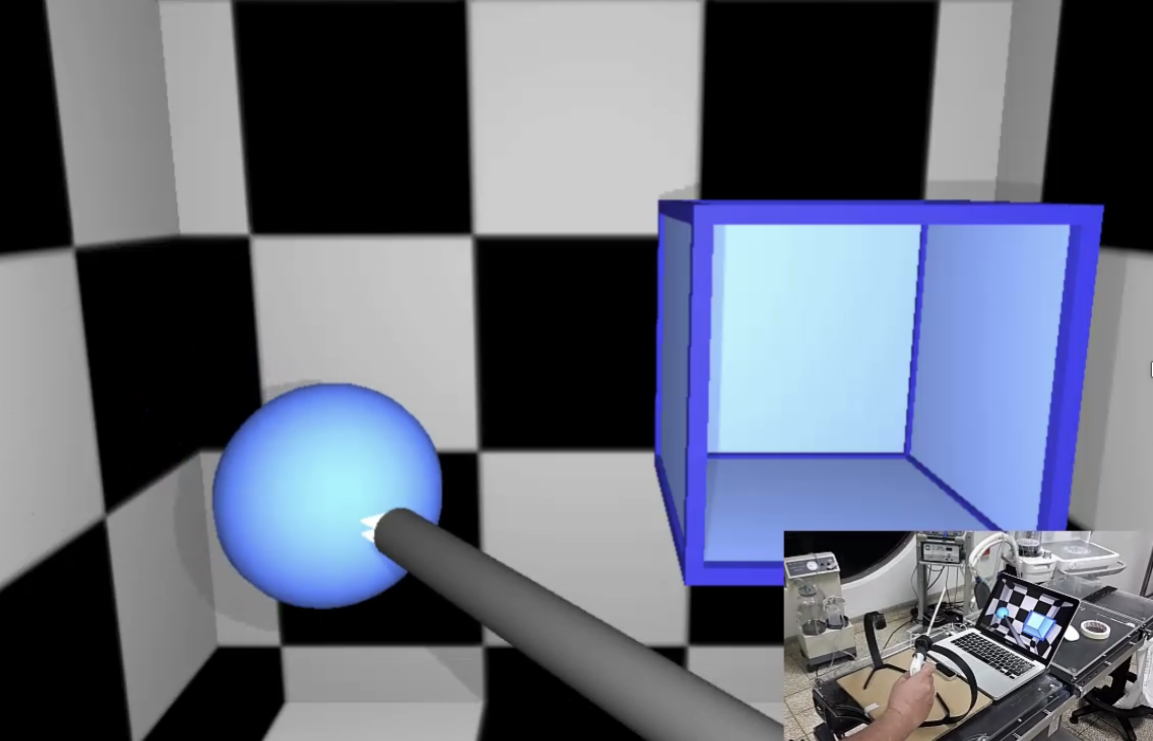
The first functional version of the virtual environment before the feedback given by surgeons with expertise in minimally invasive surgery.

**Figure 8:**
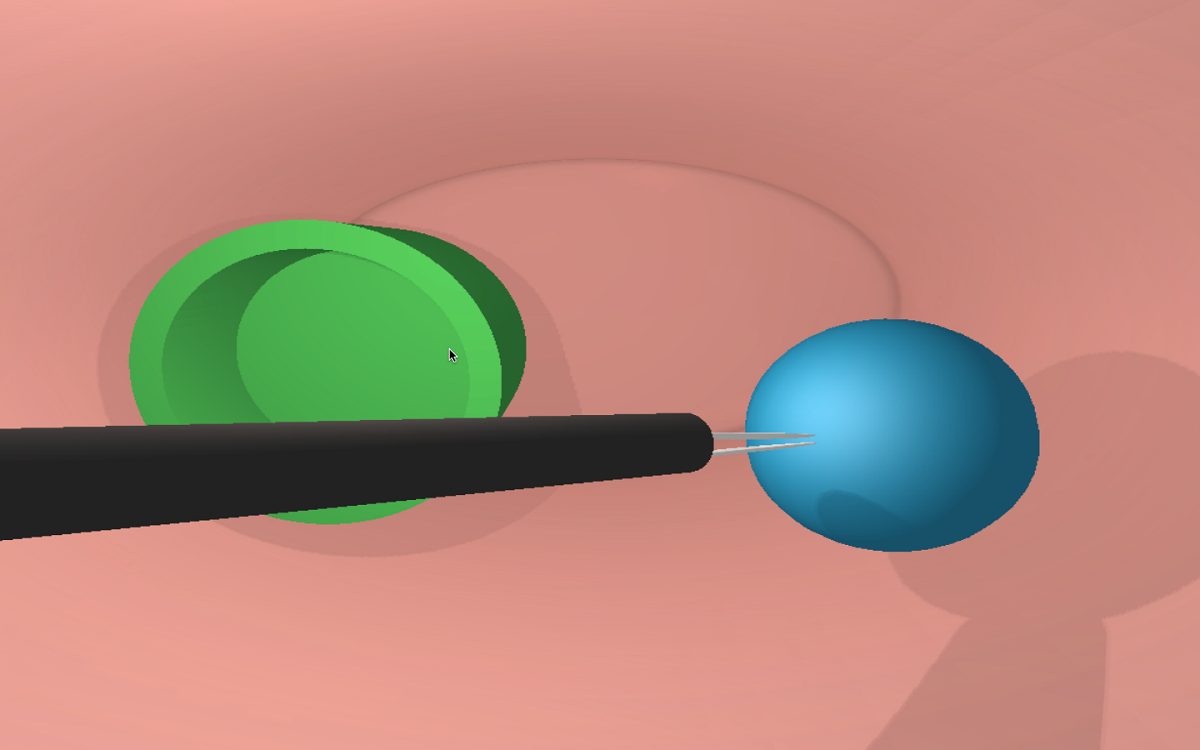
Good enough prototype of the web-based 3D virtual environment: Task 1.

Figures 6-8 show various stages in the development of the prototype for the 3D virtual environment. As in the development of the hardware elements, the 3D virtual environment design process was iterative, so that each new version of the 3D virtual environment became increasingly closer to the version considered *good enough* in terms of the design and function.

Figure 6 shows the initial attempt at the interaction between the forceps and the basic 3D virtual environment. At this stage of the design, the researchers achieved *capture* of the virtual objects by the tip of the instruments and their transfer to a virtual container (Figure 6). The second stage of development accomplished the development of the 5 tasks in a 3D virtual environment characterized by rectangular geometric shapes (Figure 7). Although the researchers did have concurrent feedback based on sounds, color changes, and a sensation of collision, at that time, the metrics had not been developed. Figure 8 shows the final *good enough* result of the 3D virtual environment. On the basis of the feedback provided by the expert surgeons, the environment was redesigned without rectangular geometric shapes, although with abstract circular shapes that were closer to the view of the body cavities during the MIS procedure.

The changes shown in Table 2 reflect the steps in the process described by Manson [16,20], where during the development of the artifact, through iterative processes of deduction and circumscription, errors were recognized in the design or function of the prototype that required further versions to be developed until the study achieved one that was considered *good enough* [16,17] and functional.

**Table 2:** Process of obtaining the good enough prototype.

Element	Initial prototype	Problem	Functional prototype	Output
MIS^a^ forceps	The shaft of the forceps is black	Difficulties in the detection of the forceps by the LMC^b^	The shaft of the forceps is white	Notable improvement in detection of the forceps by the LMC
Support devices	No support devices	Fulcrum effect not reproduced	Design of support devices	Reproduction of fulcrum effect
Mounting pad	No mounting pad	The hardware pieces (LMC and support devices) are independent, and there is no standard arrangement	Standardized integration of the pieces in the mounting pad	Physical stability of the model
Position of the LMC	Completely horizontal, 90 degree with regard to the screen	Difficulties in the detection of the forceps by the LMC	A forward 45-degree angle was applied with regard to the screen	Interference between the forceps when detected by the LMC was eliminated
First prototype of the 3D virtual environment (Figure 6)	Tests on the interaction between the forceps and the objects in the virtual environment	Difficulty for interaction between the forceps and the objects in the environment	Trial and error tests on interaction by modifying LMC and instrumental variables	Complete interaction achieved
Second prototype of the 3D virtual environment (Figure 7)	Functional environment in the 6 tasks	Quadrangular shapes in the environment	Circular shapes in the *good enough* environment (Figure 8)	An abstract 3D virtual environment with circular shapes
*Good enough* environment (Figure 8)	SQL^c^ database engine not integrated into the simulation software	A software program should be installed in addition to the simulation program	Redesign of the model and data capture and storage	Feedback and metrics complete and integrated into the SQLite Integration of the SQL database engine into the simulation software

^a^MIS: minimally invasive surgery.

^b^LMC: Leap Motion Controller.

^c^SQL: Structured Query Language.

### Phase 2: Evaluation of Fidelity to the Criterion and Subjective Validation of SIMISGEST-VR (Content Validity)

The next step in the process was the evaluation of fidelity to the criterion and the process of subjective content validity. The results are described below.

#### Demographics

A total of 30 people with an average age of 42 years (SD 2.2) participated in the study; 53% (n=16/30) were men. Those surveyed came from Colombia (n=14), Spain (n=8), Argentina (n=3), Brazil (n=2), Uruguay (n=2), and France (n=1).

Table 3 summarizes the participants’ profiles according to the level of training and experience. The residents belonged to training programs in general and pediatric surgery; one of the participants was a biomedical engineer with extensive experience in the design of devices and simulators in MIS. The vast majority (n=28/30, 93%) of participants were right-handed, 1 was left-handed (n=9/30, 3%), and the other, ambidextrous (n=9/30, 3%).

In terms of the use of video games, most (n=22/30, 73%) of those surveyed had no experience with these app; 62% (n=5/8) of those who used video games were women. Of those with experience in video games (n=8), only 1 played them weekly, while the rest played them once a month (n=3) or occasionally (n=4). The mean age of those with no experience in video games was 44 years (SD 2.7), compared with 37 years (SD 3.5) for those with experience (*P*=.16).

Only 33% (n=10/30) of the participants had experience with VR devices, and only one-third used them occasionally.

Most of the surveyed participants had previous experience with simulators. In terms of the level of operating experience, 54% (n=14/26) of the respondents with experience with simulators had an intermediate or advanced operating level, followed by those with a basic operating level (n=10/26, 38%). Among participants who had experience with simulators [26], 62% (n=16/26) had used physical simulators, 23% (n=6/26) had used hybrid simulators, and only 15% (n=4/26) had used VR simulators. The average age of those who had no experience with simulators was 40 years (SD 6.7), compared with 42 years (SD 2.4) for those with previous experience (*P*=.83).

The demographic profile questionnaire can be found in Multimedia Appendix 1.

**Table 3:** Demographic profile according to the level of experience and training (N=30).

Demographic variable		Level of experience				Level of training		
		Basic manipulation (n=3)^a^	Basic operating level (n=11)^b^	Intermediate operating level (n=8)^c^	Advanced operating level (n=8)^d^	Practicing surgeon (n=21)	Resident (n=8)	Other (n=1)^e^
Age (years), mean (SD)		26 (0.6)	40 (4.3)	43 (3.3)	49 (2.9)	47 (2.2)	27 (0.6)	49 (—^f^)
**Sex, n**								
	Male	0	3	5	8	15	0	1
	Female	3	8	3	0	6	8	0
**Do you have regular experience with video games?, n**								
	Yes	1	1	4	2	6	1	1
	No	2	10	4	6	16	6	0
**Do you have previous experience with MIS^g^ simulators?, n**								
	Yes	2	10	6	8	19	7	0
	No	1	1	2	0	3	0	1
**What type of simulator?, n**								
	Physical	2	6	3	5	12	3	1
	Hybrid and augmented reality	0	2	2	2	4	2	0
	Virtual reality	0	2	1	1	2	2	0
	No experience	1	1	2	0	3	1	0

^a^Basic manipulation of the camera and/or retraction with forceps.

^b^Basic operating level (cholecystectomy and appendectomy).

^c^Intermediate operating level (fundoplication).

^d^Advanced operating level.

^e^Other: an engineer highly experienced in the design of instruments and devices for minimally invasive surgery simulation.

^f^Not available because there was only one observation.

^g^MIS: minimally invasive surgery.

#### Evaluation of Fidelity to Criterion

Tables 4 and 5 show that there were no significant differences in the different ratings when the level of training (Table 4) or experience (Table 5) was considered.

In terms of the fidelity to the criterion, none of the respondents strongly disagreed with any of the items asked. The rating of *disagree* was given by one participant to the question about relevance, by another to the assessment of how the movements of the physical instruments were represented in the virtual environment, and 3 assigned this score when rating the fulcrum effect.

In terms of ease of use, 73% (n=22/30) and 27% (n=8/30) assigned a rating of 5 and 4, respectively. The same results were obtained when the navigation menu was assessed. With regard to the relevance of the tool as a simulator, 73% (n=22/30) assigned a score of 5 and 20% (n=6/30) assigned a score of 4.

When assessing the capacity of the physical devices to simulate the fulcrum effect, 73% (22/30) assigned a score between 4 and 5, 17% (n=5/30) assigned a score of 3, and 10% (n=3/30) assigned a score of 2. For this last rating, in terms of the level of training, 2 were practicing surgeons and 1 was a resident, whereas in terms of the level of experience, one corresponded to basic manipulation, one to intermediate operating level, and another to advanced level.

In terms of how the movements of the forceps were represented in the virtual environment, 73% (n=22/30) rated this as 4 or 5, 23% (n=7/30) assigned a score of 3, and only one of the participants (n=9/30, 3%) assigned a score of 2 (level of training=practicing surgeon and level of experience=intermediate).

When assessing how appropriately the tool simulates the movements of MIS, 83% (n=25/30) rated the question as 4 or 5. All respondents (n=30/30, 100%) rated the design as attractive, with scores of 4 or 5. Almost all surveyed respondents (n=29/30, 97%) assigned ratings of 4 or 5 to the innovation factor, the capacity to provide feedback, and to the question of whether the latter was adequate.

The fidelity to the criterion study questions can be found in Multimedia Appendix 1. The fidelity to the criterion study result tables can be found in Multimedia Appendix 2.

**Table 4:** Fidelity to the criterion and content validity according to the level of training.

Variable		Resident (n=8)					Practicing surgeon (n=21)					Other^a^ (n=1)					*P* value
		Median		IQR		Median		IQR		Median			IQR				
**Fidelity to the criterion^b^**																	
	Ease of use		5		4.5-5		5		4-5		5			5-5		.88	
	Navigation menu		5		5-5		5		4-5		5			5-5		.62	
	Relevance as a learning tool		5		4-5		5		5-5		5			5-5		.73	
	Fulcrum effect		3.5		3-4		5		4-5		4			4-4		.13	
	Representation of the physical forceps in the virtual environment		4		3-5		4		4-5		5			5-5		.56	
	Simulation of the movements in MIS^c^		4		4-4		4		4 - 5		5			5-5		.18	
	Innovation		5		4.5-5		5		5-5		5			5-5		.90	
	Graphic design		4.5		4-5		5		4-5		5			5-5		.69	
	Feedback		5		4-5		5		5-5		5			5-5		.79	
	Relevance of the feedback		4		4-5		5		4-5		5			5-5		.43	
**Content validity^d^**																	
	Hand-eye coordination		4.5		4-5		5		4-5		5			5-5		.66	
	Depth perception		4		3.5-5		5		4-5		5			5-5		.41	
	Basic psychomotor skills learning		4.5		4-5		5		5-5		5			5-5		.42	
	Basic steps of MIS		4		4-5		5		4-5		5			5-5		.64	
	Metrics		4		3-5		4		4-5		5			5-5		.43	
	Ubiquitous learning		4		4-5		5		4-5		5			5-5		.31	
**Tasks^e^**																	
	Task 1		3.5		3-4		4		4-5		5			5-5		.19	
	Task 2		4		4-4.5		4		3-5		5			5-5		.41	
	Task 3		4		3.5-4		4		3-5		5			5-5		.40	
	Task 4		4		3-5		5		4-5		2			2-2		.21	
	Task 5		4.5		4-5		5		4-5		5			5-5		.65	
	Task 6		4		4-4		5		4-5		5			5-5		.02	

^a^Other: An engineer highly experienced in the design of instruments and devices for minimally invasive surgery simulation.

^b^For fidelity to the criterion questions, see Multimedia Appendix 1.

^c^MIS: minimally invasive surgery.

^d^For content validity questions, see Multimedia Appendix 1.

^e^For task descriptions, see Table 1.

**Table 5:** Fidelity to the criterion and content validity according to the level of experience.

Variable		Basic manipulation (n=3)				Basic operating level (n=11)				Intermediate operating level (n=8)				Advanced operating level (n=8)				*P* value		
		Median		IQR		Median		IQR		Median		IQR		Median		IQR				
**Fidelity to the criterion^a^**																				
	Ease of use		5		4-5		5		4-5		5		4.5-5		4.5		4-5			.84
	Navigation menu		5		4-5		5		5-5		5		4.5-5		4.5		4-5			.51
	Relevance as a learning tool		5		2-5		5		4-5		5		5-5		5		4-5			.83
	Fulcrum effect		4		2-5		4		3-5		5		4-5		4		4-5			.66
	Representation of the physical forceps in the virtual environment		5		3-5		4		4-5		4		3.5-5		4.5		3-5			.96
	Simulation of the movements in MIS^b^		4		4-4		4		4-5		4.5		3.5-5		4		3.5-4.5			.70
	Innovation		5		3-5		5		5-5		5		4.5-5		5		4.5-5			.95
	Graphic design		4		4-5		5		4-5		5		4.5-5		4		4-5			.41
	Feedback		5		4-5		5		4-5		5		5-5		4.5		4-5			.42
	Relevance of the feedback		4		4-5		5		4-5		5		4.5-5		4.5		4-5			.66
**Content validity^c^**																				
	Hand-eye coordination		4		4-5		5		4-5		5		5-5		4.5		4-5			.77
	Depth perception		5		4-5		5		4-5		5		4-5		4.5		4-5			.95
	Basic psychomotor skills learning		4		3-5		5		4-5		5		5-5		5		4-5			.45
	Basic steps of MIS		4		4-5		5		4-5		5		4.5-5		4		4-4.5			.33
	Metrics		4		3-5		4		3-5		4.5		4-5		4.5		4-5			.75
	Ubiquitous learning		4		4-5		5		4-5		5		5-5		4.5		4-5			.46
**Tasks^d^**																				
	Task 1		3		3-5		4		3-5		4		3.5-5		4		3.5-4.5			.88
	Task 2		4		4-5		4		4-5		4		3.5-5		3.5		2-5			.76
	Task 3		4		2-5		4		3-4		4.5		3.5-5		4		3.5 – 5			.76
	Task 4		2		2-5		4		4-5		5		5-5		4		3.5-5			.18
	Task 5		4		4-5		5		4-5		5		4.5-5		4.5		4-5			.70
	Task 6		4		4-4		4		4-5		5		5-5		4.5		4-5			.12

^a^For fidelity to the criterion questions, see Multimedia Appendix 1.

^b^MIS: minimally invasive surgery.

^c^For content validity questions, see Multimedia Appendix 1.

^d^For task descriptions, see Table 1.

#### Content Validity

Table 6 summarizes the sources of validity evidence for the MIST-VR and the studies that have demonstrated such validity.

With regard to content validity, none of the items evaluated for the training capacity were rated as 1, although, in the case of hand-eye coordination by a practicing surgeon with an advanced operating level and the depth perception by a practicing surgeon with an intermediate operating level, the hand-eye coordination and depth perception were rated as 2. Almost all of those surveyed (n=28/30, 93%) rated the hand-eye coordination as 4 or 5, while 87% (n=26/30) gave this score for depth perception.

The highest-rated item was the one that considered that the prototype could be a solution for ubiquitous learning in MIS: 100% (n=30/30) of those surveyed rated it as 4 or 5. With regard to the evaluation of the metrics, 17% (n=5/30) of those surveyed rated them as 3, while the remaining participants (n=25/30) rated them as 4 or 5.

Almost all respondents (n=29/30, 97%) considered that the SIMISGEST-VR enables learning of basic psychomotor skills in MIS, with ratings of 4 and 5; whereas, 93% (n=28/30) agreed that the tasks reflect the basic steps of a minimally invasive procedure, with ratings of 4 and 5.

An analysis of the evaluation of the tasks, in general, showed that the following were rated between 4 and 5: Task 1 received this rating from 70% (n=21/30) of those interviewed; Task 2 from 77% (n=23/30); Task 3 from 73% (n=22/30); Task 4 from 77% (n=23/30); and Task 5 and Task 6 from 90% (n=27/30) of the participants.

For Task 6 (Table 4), a lower score was assigned by individuals with lower levels of training (*P*=.02).

The content validity study questions can be found in Multimedia Appendix 1. The tables of results of the content validity study can be found in Multimedia Appendix 3.

**Table 6:** Sources of validity evidence for the minimally invasive surgery training–virtual reality.

Source of validity evidence	Studies
Content evidence	[9,10,43,46-51]
Internal structure	[41,43,48,52-62]
Relationship to other variables	[4,10,12,13,37,43,47,50-57,60,63-116]
Consequences	[4,11,13,43,47,49,50,54,56,57,59,69,71,73,78,79,92,104,107,108,114,117-123]

## Discussion

### Principal Findings

Simulation as a tool for learning psychomotor skills in MIS has become a new model for education in surgery. The use of human or animal cadavers is becoming increasingly controversial for learning surgical maneuvers [124,125], resulting in an immense growth of simulation using virtual environments as a tool for learning psychomotor skills in MIS and for the simulation of full surgical procedures [5,126].

Simulators for psychomotor skills learning in MIS are classified into mechanical, hybrid/augmented reality, or VR [6,7]. Devices for gesture-based human-computer interaction are a new way of interacting with virtual environments. This study’s simulator presents a new form of gesture-based simulation that is portable, low-cost, and enables ubiquitous learning and preoperative warm-up [14,127,128].

### Development of Gesture-Mediated Simulator for Minimally Invasive Surgery—Virtual Reality

The development of SIMISGEST-VR was based on DBR principles. It was a *pragmatic* process because the researchers tried to resolve the problems of portability and the high cost of simulators for learning psychomotor skills in MIS. It was *grounded* in both theory and the real-world context, as we designed a functional simulator based on theories on simulation-based surgical skills training. It was *interactive*, in that during the simulator design stage, a *good enough* prototype was obtained through the participation of an interdisciplinary team (pediatric surgery, systems engineering, graphic design, and experts in education and psychology), as well as the comments and feedback provided by experts in MIS during the subjective validation study. Finally, the process was *iterative*, in that a process of analysis, design, evaluation, and redesign was applied (Table 1) until a *good enough* protocol was obtained that could be subjected to validation studies [129].

To develop this study’s 3D virtual environment, the researchers adopted the principle of low fidelity, given that the model is envisaged for basic psychomotor skills learning. The term fidelity refers to the extent to which a simulation imitates reality (in the case of surgical simulation, the anatomy) and is considered a critical variable in the design of simulators. However, this statement is not necessarily completely true, as for novice learners, low-fidelity models that reproduce the essential constructs of a procedure allow a faster and more cost-effective learning curve to be achieved [35,130]. Thus, in the field of simulation in aviation, simple images reduce the learner’s confusion when learning basic skills [131], while experts benefit from higher fidelity simulations [33,132].

The tasks were adapted from the MIST-VR, which is the only laparoscopic VR trainer that can act as a standard because it is the sole surgical VR system that has been reasonably validated [4,11,65,133]. MIST-VR has been shown to allow the learning of basic skills that can be transferred to the surgical environment at a more reasonable cost [4,11,52,73,134].

#### Metrics

Performance evaluation is a fundamental part of the learning process and is essential for certification. To obtain an objective evaluation of performance, the simulator should define metrics that must be valid, accurate, and relevant in terms of the procedure that is being taught. Evaluation using metrics and effective feedback are the most important elements of effective learning in a simulation environment [7]. Metrics allow an objective measure of motor performance to be obtained and enable the learning progress to be compared and tracked [10,43,44]. Accordingly, if the metrics lack sensitivity and validity, training on simulators will not be optimal and the learning will be affected [135]. In SIMISGEST-VR, the metrics were determined by time, the efficiency of movement, economy of diathermy, and error. This was an iterative process involving several pilot studies and modifications to the tasks and their metrics based on feedback provided by surgeons and education experts.

#### Feedback

Feedback is essential [136,137]. Training on a simulator should have 3 purposes: (1) to improve performance; (2) to make the performance consistent; and (3) to reduce the number of errors [57]. The metrics and feedback are essential for achieving these objectives. On the SIMISGEST-VR, the study adopted 3 types of feedback: (1) concurrent, which is provided while the task is being performed; (2) immediate, when the exercise is finished; and (3) terminal, which shows the final score when all the tasks have been completed [136,138-140].

#### Hardware

The design of the hardware components aimed to simulate the movements made by the surgeon during MIS. These movements are defined by the physical characteristics of the devices and, therefore, require the design of mechanical support devices that simulate the fulcrum effect (entry portals), add friction to the movements of the forceps, and limit arm movement during the performance of the tasks without interfering with the reading of the instrument movements by the LMC [141,142]. The portability and low cost were also taken into account.

#### Cost of Gesture-Mediated Simulator for Minimally Invasive Surgery-Virtual Reality

The VR or augmented VR simulators currently available in the market are not portable, and their cost ranges from US $2000 to US $100,000 (with annual maintenance costs of US $25,000) for a haptic VR simulator. The LMC costs approximately US $130, plus a further US $70 for the hardware elements, adding up to a total cost of approximately US $200 for the SIMISGEST-VR, software costs excluded.

#### Subjective Validation of Gesture-Mediated Simulator for Minimally Invasive Surgery–Virtual Reality

The second aim of this study was to evaluate fidelity to the criterion and a content validity study. Validity refers to the quality of the inferences, claims, or decisions taken from the scores given by an instrument, not the instrument itself. Validation for its part is a process through which the evidence that supports the quality, significance, and utility of the decisions and inferences that can be made from the scores provided by the instrument is drawn together and evaluated [143]. Validity is not an all-or-nothing statement, as it reflects a gradual appraisal that depends on the purpose of the measurement and the proper interpretation of the results. Validity is also not in itself a characteristic of the system, but the appropriate interpretation and use of the measurement results of the system. A single instrument may be used for many different purposes, and the resulting scores may be more valid for one purpose than for another [133].

#### Study of Fidelity to Criterion

Although it has been deemed that *face validity* should no longer be considered a type of validity or used as a term in validation studies [144,145], its assessment is extremely important during the design phase of any evaluation device [146,147]. Therefore, the use of an alternative term to denominate this type of evaluation has been suggested: *fidelity to the criterion* [148]. Despite such warnings, it is very striking to find that the term *face validity* is still being used in published literature on simulation in surgery [149,150].

Fidelity to the criterion evaluates to what point the test reflects the real-life situation, whether the simulator represents what it is supposed to represent (the realism of the simulator) or the extent to which a questionnaire or other measurement reflects the variable to be measured [125,151,152]. In the case of DBR, it is used in the initial phase of the construction of the test. The surveys that assess fidelity to the criterion feedback into the iterative design process, which allows the *good enough* prototype to be obtained [153]. Fidelity to the criterion is evaluated by experts and novices called referents [154,155].

In this study, the evaluation of fidelity to the criterion provided feedback on the initial design, and this was how the 3D virtual environment was redesigned until a *good enough* prototype was obtained. The quality of this evaluation is improved systematically when structured questionnaires and Likert scales are applied [154].

In all the items evaluated for fidelity to the criterion, most of those surveyed assigned scores of 4 or 5. There were no significant differences between the expert and referent groups (level of training) when rating fidelity to the criterion. The lowest scores were obtained for the item about the relevance (n=9/30, 3% of participants), the representation of the movements of the physical forceps in the virtual environment (n=9/30, 3%), and for the fulcrum effect (n=3/30, 10%).

#### Evidence Based on Test Content

The latest standards on validity and validation refer to sources of validity evidence, rather than distinct types of validity. Validity therefore refers to the degree to which the evidence and theory support the interpretations of test scores for the proposed uses of tests [156,157].

Evidence based on test content is an issue of representation and may be obtained from an analysis of the relationship between test content and the construct that is intended to be measured. In this study, the test content refers to the simulator’s 6 specific tasks. Evidence can be obtained from logical or empirical analyses of how test content represents domain content and of the relevance of domain content to the proposed interpretation of test scores. Evidence may also come from experts’ opinions on the relationship between the different test items and the construct when assessing whether the test contains the meaningful steps, skills, and materials used in the actual procedure [158] and determines whether the simulator can realistically teach what it is supposed to represent [159].

The question is, does the simulator realistically teach what it should teach? In other words, does the instrument represent all the ways in which it can be used to measure the content of a given construct? [160]. In summary, evidence based on test content judges the appropriateness of the simulator as a teaching modality or as a training tool within the domain that it seeks to measure [31,151,152].

This type of validation is highly recommended in the practice of DBR during the design phase of the *good enough* prototype. Content validity can be obtained from a literature review, an expert review, using content validity rates, and *Q sorting* [161].

The tasks within the surgical simulation should fulfill 3 criteria: objectivity, clarity, and completeness. To be objective, the definition of the task should refer to observable characteristics of the behavior; for it to be clear, the task should be unambiguous so that it can be read, understood, and reproduced equally by different observers; and finally, to meet the criterion of completeness, the definition of the task should delineate its start and end and make it clear when it was completed [162].

In this study, the 6 skill tasks were chosen for two main reasons: (1) these tasks are well-validated in many clinical studies [4,10,82,117] using the MIST-VR (Table 6); and (2) they contain laparoscopic skills and techniques that are usually present in many laparoscopic procedures (Table 1).

The vast majority of study participants considered that the SIMISGEST-VR was a useful tool for the development of hand-eye coordination and depth perception, with ratings of 4 and 5 on the Likert scale. Similarly, there was consensus about the capacity of the simulator to teach basic psychomotor skills and to reflect the basic steps in MIS. All the respondents considered the metrics to be adequate and envisaged that the simulator could become a solution to achieve ubiquitous learning of basic psychomotor skills in MIS.

In terms of the specific rating for each of the 6 tasks, this varied between 3.97 and 4.53. The participants considered all the items of the SIMISGEST-VR training system as good to excellent.

Finally, the study of fidelity to the criterion and content validity must be proven in the design stage of the artifact, before the criterion (concurrent and predictive) and construct validity (convergent and discriminative) can be confirmed. The evaluation of fidelity to the criterion, although somewhat subjective, is a necessary assessment during the initial phase of any high-stakes test construction and in this study, within the context of DBR, in the design phase of prototypes that will give a *good enough* prototype as a result [154,158,163]. In conclusion, the results of the study of fidelity to the criterion and content-related validity evidence showed overall positive scores.

#### Threats to Validity

The *Hawthorne effect* occurs when the opinion may be influenced by the attention paid to the respondent during his or her performance with the simulator, which may contribute to the occurrence of favorable responses or scores. This effect can be ameliorated by paying equal attention to each respondent. In addition, the *Pygmalion effect* occurs when the enthusiasm shown by the developers or because the novelty of the artifact affects the opinion of the respondent; the referent group is more prone to this latter effect [154,164]. In this study, the SIMISGEST-VR developer conducted the interviews and applied the Likert scale questionnaires; this may have influenced the ratings assigned by the participants (*Hawthorne effect)*.

Regarding the representation of the construct, in this study there was an underrepresentation—when compared with the learning models based on training boxes—referring to the *cut* skill of the *basic psychomotor skills* construct, which was because of technical reasons associated with the LMC (construct context). There was no overrepresentation of the construct [165].

### Limitations

There are, however, limitations to this study. The sample size of this study was one of availability and, for the simulator to be portable and allow ubiquitous learning, the researchers disregarded some ergonomic principles applied to MIS [166,167]. Further research will be conducted using new motion metrics, new skill tasks, and the development of the web-based virtual environment for download as an app. In addition, the researchers of this study are working on the development of different difficulty levels for each exercise.

### Future Work

The researchers of this study are currently conducting another study to show validity evidence for the *good enough* prototype described in this paper, using the new framework for validation in education [168,169]. This new study is expected to verify the sources of validity evidence for the internal structure, relationships between variables, and test consequences.

Once the metrics and the results of the performance scores have been validated as a useful tool for learning basic psychomotor skills in MIS, a model will be obtained to enable ubiquitous learning in MIS and preoperative warm-up by using the 3D reconstruction of patient images [14]. Studies conducted in this area have demonstrated that, generally speaking, preoperative warm-up exercises performed for at least 15 min before the procedure improve the surgeon's handling of soft tissue during cholecystectomy [170], bimanual skill, efficiency and smoothness of movement, and depth perception, at the same time as mistakes and operating time are reduced [171-177].

The large size and elevated costs of VR simulators currently available in the market prohibit their use in the operating theater. A portable, low-cost simulation solution, such as the SIMISGEST-VR, would allow surgeons to perform preoperative warm-up exercises anytime, anywhere (ubiquitous learning). In addition, the researchers aim to enable a surgeon to perform warm-up exercises based on 3D reconstructions of preoperative images of a specific patient, thus, practicing the procedure before performing the actual surgery. This could take place the night before in the surgeon's home or the operating theater on the day of the surgery [178-183]

### Conclusions

This study demonstrated the feasibility of a portable, low-cost, gesture-based, functional simulator (SIMISGEST-VR) for learning basic psychomotor skills in MIS.

The results of the evaluation of fidelity to the criterion and content validity showed overall positive scores, which indicates that the SIMISGEST-VR would be acceptable to both the expert group and referent group as a training and learning device (including at home) to achieve ubiquitous learning in MIS.

The participants in the study agreed that content validity was acceptable, accurate, and representative in the field of basic psychomotor skills learning in MIS.

## Appendix

Multimedia Appendix 1 Application forms of the demographic survey, fidelity to the criterion, and content validity surveys.

Multimedia Appendix 2 Results of the fidelity to the criterion survey.

Multimedia Appendix 3 Results of the content validity survey.

## Abbreviations

MIS: minimally invasive surgery
MIST-VR: minimally invasive surgery training—virtual reality
LMC: Leap Motion Controller
DBR: design-based research
SIMISGEST-VR: gesture-mediated simulator for minimally invasive surgery—virtual reality
