# Supplementary material for: Use of a Low-Cost Portable 3D Virtual Reality Gesture-Mediated Simulator for Training and Learning Basic Psychomotor Skills in Minimally Invasive Surgery: Development and Content Validity Study
Source: J Med Internet Res. 2020 Jul 14;22(7):e17491. doi: 10.2196/17491 (PMC7388055; doi:10.2196/17491)
Supplement: Multimedia Appendix 1 [file jmir_v22i7e17491_app1.docx]

**Appendix 1. Application forms of the demographic survey, fidelity to the criterion and content validity surveys.**

1.1 Demographic survey.

Name ______________________________________________________

Age ______

Sex ______

Nationality _______________

Dominant hand: Right___ Left___ Ambidextrous___

Do you have previous experience with simulators in minimally invasive surgery? **YES __ NO __**

If YES, what type of simulator?

- Physical ___
- Hybrid/augmented virtual reality ___
- Virtual reality ___

Do you have regular experience with video games? **YES __ NO __**

If YES, how often do you play video games?

| Daily | Weekly | Monthly | Occasionally |
| --- | --- | --- | --- |

Have you had previous experience with virtual reality devices **YES ___ NO ___**

If YES, how often do you perform activities with virtual/augmented reality devices?

| Daily | Weekly | Monthly | Occasionally |
| --- | --- | --- | --- |

Select the most appropriate answer regarding your level of education

1. Undergraduate student
2. Surgical resident
3. Practicing surgeon
4. Other. Please specify. ___________________

Select which of these options describes your level of experience in laparoscopic surgery:

1. None
2. Basic manipulation of the camera and/or retraction with forceps
3. Basic operating level (cholecystectomy, appendectomy)
4. Intermediate operating level (fundoplication)
5. Advanced level

Please, tell us which technology or tools do you regularly use:

|  | At work | Personal use | Work and personal use | I don’t use this technology |
| --- | --- | --- | --- | --- |
| Mobile phone with internet connection |  |  |  |  |
| Desktop computer |  |  |  |  |
| Laptop computer |  |  |  |  |
| Smartphone (iPhone, Samsung Galaxy, etc.) |  |  |  |  |
| Tablet |  |  |  |  |

# 1.2 Fidelity to the criterion survey.

| **Fidelity to the criterion** | | | | | |
| --- | --- | --- | --- | --- | --- |
|  | 1. Strongly disagree | 2. Disagree | 3. Neither agree nor disagree | 4. Agree | 5. Strongly agree |
| Was the tool easy to use? |  |  |  |  |  |
| Was the navigation menu of the tool user-friendly? |  |  |  |  |  |
| Do you consider that the tool is relevant as a simulator for basic psychomotor skills training in laparoscopic surgery? |  |  |  |  |  |
| Do the physical devices of the tool give an adequate sensation of the fulcrum effect? |  |  |  |  |  |
| Did you have the sensation that the movements you made with the physical instrument were represented in the virtual environment? |  |  |  |  |  |
| Does the tool properly simulate the movements of laparoscopic surgery? |  |  |  |  |  |
| Do you consider this idea to be innovative? |  |  |  |  |  |
| Do you consider the design to be attractive? |  |  |  |  |  |
| Do you consider that the tool has the ability to provide feedback? |  |  |  |  |  |
| Did you feel that the feedback provided was adequate? |  |  |  |  |  |
| Please make a final comment about the tool |  | | | | |

1.3 Content validity survey

| **Content validity** | | | | | |
| --- | --- | --- | --- | --- | --- |
|  | 1. Strongly disagree | 2. Disagree | 3. Neither agree nor disagree | 4. Agree | 5. Strongly agree |
| **Training capacity** |  |  |  |  |  |
| Do you consider that the exercises allow the learning of hand-eye coordination? |  |  |  |  |  |
| Do you consider that the exercises allow the learning of depth perception? |  |  |  |  |  |
| Do you consider that the virtual environment allows the learning of basic psychomotor skills in laparoscopic surgery? |  |  |  |  |  |
| Do you consider that the virtual environment reflects the basic steps of any laparoscopic procedure? |  |  |  |  |  |
| Do you consider that the performance metrics provided (time, error, efficiency of movement, economy of diathermy) are adequate? |  |  |  |  |  |
| Do you consider that the prototype could become a solution for ubiquitous learning of basic psychomotor skills in laparoscopic surgery? |  |  |  |  |  |
| **Tasks** |  |  |  |  |  |
| Task 1. Grip and placement. Does it reflect the grasping and retraction of a tissue into a given position? |  |  |  |  |  |
| Task 2. Transfer and place. Does it reflect the manipulation of a needle in an intracorporeal suture? |  |  |  |  |  |
| Task 3. Cross. Does it reflect exploration of the small intestine? |  |  |  |  |  |
| Task 4. Removal and introduction. Does it reflect the removal and introduction of the laparoscopic instruments? |  |  |  |  |  |
| Task 5. Diathermy. Does it reflect the cauterization of a blood vessel? |  |  |  |  |  |
| Task 6. Manipulation and diathermy. Does it reflect cauterization of the gallbladder bed? |  |  |  |  |  |
