# Supplementary material for: Use of a Low-Cost Portable 3D Virtual Reality Gesture-Mediated Simulator for Training and Learning Basic Psychomotor Skills in Minimally Invasive Surgery: Development and Content Validity Study
Source: J Med Internet Res. 2020 Jul 14;22(7):e17491. doi: 10.2196/17491 (PMC7388055; doi:10.2196/17491)
Supplement: Multimedia Appendix 2 [file jmir_v22i7e17491_app2.docx]

**Appendix 2.** **Results of the fidelity to the criterion survey.**

2.1 Fidelity to the criterion.

| **Fidelity to the criterion** | | | | | |
| --- | --- | --- | --- | --- | --- |
|  | **1** | **2** | **3** | **4** | **5** |
| Was the tool easy to use? | 0 | 0 | 0 | 8 | 22 |
| Was the navigation menu of the tool user-friendly? | 0 | 0 | 0 | 8 | 22 |
| Do you consider that the tool is relevant as a simulator for basic psychomotor skills training in laparoscopic surgery? | 0 | 1 | 1 | 6 | 22 |
| Do the physical devices of the tool give an adequate sensation of the fulcrum effect? | 0 | 3 | 5 | 9 | 13 |
| Did you have the sensation that the movements you made with the physical instrument were represented in the virtual environment? | 0 | 1 | 7 | 9 | 13 |
| Does the tool properly simulate the movements of laparoscopic surgery? | 0 | 0 | 5 | 14 | 11 |
| Do you consider this idea to be innovative? | 0 | 0 | 1 | 6 | 23 |
| Do you consider the design to be attractive? | 0 | 0 | 0 | 12 | 18 |
| Do you consider that the tool has the ability to provide feedback? | 0 | 0 | 1 | 7 | 22 |
| Did you feel that the feedback provided was adequate? | 0 | 0 | 1 | 11 | 18 |

2.2 Fidelity to the criterion according to level of training

| **Fidelity to the criterion** | **Total  (n = 30)** | **Residents  (n = 8)** | **Practicing surgeon  (n = 21)** | **Other  (n = 1)** | **p** |
| --- | --- | --- | --- | --- | --- |
| Was the tool easy to use? | 4.73 | 4.75 | 4.71 | 5.00 | 0.819 |
| Was the navigation menu of the tool user-friendly? | 4.73 | 4.88 | 4.67 | 5.00 | 0.448 |
| Do you consider that the tool is relevant as a simulator for basic psychomotor skills training in laparoscopic surgery? | 4.63 | 4.38 | 4.71 | 5.00 | 0.599 |
| Do the physical devices of the tool give an adequate sensation of the fulcrum effect? | 4.07 | 3.50 | 4.29 | 4.00 | 0.108 |
| Did you have the sensation that the movements you made with the physical instrument were represented in the virtual environment? | 4.13 | 4.00 | 4.14 | 5.00 | 0.526 |
| Does the tool properly simulate the movements of laparoscopic surgery? | 4.20 | 3.88 | 4.29 | 5.00 | 0.140 |
| Do you consider this idea to be innovative? | 4.73 | 4.63 | 4.76 | 5.00 | 0.838 |
| Do you consider the design to be attractive? | 4.60 | 4.50 | 4.62 | 5.00 | 0.607 |
| Do you consider that the tool has the ability to provide feedback? | 4.70 | 4.63 | 4.71 | 5.00 | 0.682 |
| Did you feel that the feedback provided was adequate? | 4.57 | 4.38 | 4.62 | 5.00 | 0.327 |

2.3 Fidelity to the criterion according to level of experience

| **Fidelity to the criterion** | **Total  (n = 30)** | **Basic manipulation  (n = 3)** | **Basic operating level  (n = 11)** | **Intermediate operating level  (n = 8)** | **Advanced operating level  (n = 8)** | **p** |
| --- | --- | --- | --- | --- | --- | --- |
| Was the tool easy to use? | 4.73 | 4.67 | 4.64 | 4.88 | 4.75 | 0.710 |
| Was the navigation menu of the tool user-friendly? | 4.73 | 4.67 | 4.91 | 4.75 | 4.500 | 0.271 |
| Do you consider that the tool is relevant as a simulator for basic psychomotor skills training in laparoscopic surgery? | 4.63 | 4.0 | 4.64 | 4.88 | 4.63 | 0.694 |
| Do the physical devices of the tool give an adequate sensation of the fulcrum effect? | 4.07 | 3.67 | 3.91 | 4.38 | 4.13 | 0.619 |
| Did you have the sensation that the movements you made with the physical instrument were represented in the virtual environment? | 4.13 | 4.33 | 4.18 | 4.00 | 4.13 | 0.961 |
| Does the tool properly simulate the movements of laparoscopic surgery? | 4.20 | 4.00 | 4.36 | 4.25 | 4.00 | 0.642 |
| Do you consider this idea to be innovative? | 4.73 | 4.33 | 4.82 | 4.75 | 4.75 | 0.885 |
| Do you consider the design to be attractive? | 4.60 | 4.33 | 4.73 | 4.75 | 4.38 | 0.269 |
| Do you consider that the tool has the ability to provide feedback? | 4.70 | 4.67 | 4.64 | 5.00 | 4.50 | 0.196 |
| Did you feel that the feedback provided was adequate? | 4.57 | 4.33 | 4.64 | 4.75 | 4.38 | 0.539 |

* Basic manipulation of the camera and/or retraction with forceps

& Basic operating level (cholecystectomy, appendectomy)

+ Intermediate operating level (fundoplication)

^ Advanced operating level

2.4 Fidelity to the criterion vs. experience and training levels.

|  | **Level of experience** | | | | **Level of training** | | |
| --- | --- | --- | --- | --- | --- | --- | --- |
|  | **Basic manipulation  (n = 3)** | **Basic operating level  (n = 11)** | **Intermediate operating level  (n = 8)** | **Advanced operating level  (n = 8)** | **Practicing surgeon  (n = 21)** | **Resident  (n = 8)** | **Other  (n = 1)** |
| *Was the tool easy to use?* | | | | | | | |
| 1 | 0 | 0 | 0 | 0 | 0 | 0 | 0 |
| 2 | 0 | 0 | 0 | 0 | 0 | 0 | 0 |
| 3 | 0 | 0 | 0 | 0 | 0 | 0 | 0 |
| 4 | 1 | 4 | 1 | 2 | 6 | 2 | 0 |
| 5 | 2 | 7 | 7 | 6 | 15 | 6 | 1 |
| *The menu of navigation was friendly?* | | | | | | | |
| 1 | 0 | 0 | 0 | 0 | 0 | 0 | 0 |
| 2 | 0 | 0 | 0 | 0 | 0 | 0 | 0 |
| 3 | 0 | 0 | 0 | 0 | 0 | 0 | 0 |
| 4 | 1 | 1 | 2 | 4 | 7 | 1 | 0 |
| 5 | 2 | 10 | 6 | 4 | 14 | 7 | 1 |
| *Do you consider that the tool is relevant as a simulator for basic psychomotor skills training in MIS?* | | | | | | | |
| 1 | 0 | 0 | 0 | 0 | 0 | 0 | 0 |
| 2 | 1 | 0 | 0 | 0 | 0 | 1 | 0 |
| 3 | 0 | 1 | 0 | 0 | 1 | 0 | 0 |
| 4 | 0 | 2 | 1 | 3 | 4 | 2 | 0 |
| 5 | 2 | 8 | 7 | 5 | 16 | 5 | 1 |
| *Do the physical devices of the tool give an adequate sensation of the fulcrum effect?* | | | | | | | |
| 1 | 0 | 0 | 0 | 0 | 0 | 0 | 0 |
| 2 | 1 | 0 | 1 | 1 | 2 | 1 | 0 |
| 3 | 0 | 5 | 0 | 0 | 2 | 3 | 0 |
| 4 | 1 | 2 | 2 | 4 | 5 | 3 | 1 |
| 5 | 1 | 4 | 5 | 3 | 12 | 1 | 0 |
| *Did you have the feeling that the movements you did with the physical instrument were represented in the virtual environment?* | | | | | | | |
| 1 | 0 | 0 | 0 | 0 | 0 | 0 | 0 |
| 2 | 0 | 0 | 1 | 0 | 1 | 0 | 0 |
| 3 | 1 | 2 | 1 | 3 | 4 | 3 | 0 |
| 4 | 0 | 5 | 3 | 1 | 7 | 2 | 0 |
| 5 | 2 | 4 | 3 | 4 | 9 | 3 | 1 |
| *Does the tool properly simulate the movements of MIS?* | | | | | | | |
| 1 | 0 | 0 | 0 | 0 | 0 | 0 | 0 |
| 2 | 0 | 0 | 0 | 0 | 0 | 0 | 0 |
| 3 | 0 | 1 | 2 | 2 | 4 | 1 | 0 |
| 4 | 3 | 5 | 2 | 4 | 7 | 7 | 0 |
| 5 | 0 | 5 | 4 | 2 | 10 |  | 1 |
| *Do you consider this idea to be innovative?* | | | | | | | |
| 1 | 0 | 0 | 0 | 0 | 0 | 0 | 0 |
| 2 | 0 | 0 | 0 | 0 | 0 | 0 | 0 |
| 3 | 1 | 0 | 0 | 0 | 0 | 1 | 0 |
| 4 | 0 | 2 | 2 | 2 | 5 | 1 | 0 |
| 5 | 2 | 9 | 6 | 6 | 16 | 6 | 1 |
| *Do you consider the design attractive?* | | | | | | | |
| 1 | 0 | 0 | 0 | 0 | 0 | 0 | 0 |
| 2 | 0 | 0 | 0 | 0 | 0 | 0 | 0 |
| 3 | 0 | 0 | 0 | 0 | 0 | 0 | 0 |
| 4 | 2 | 3 | 2 | 5 | 8 | 4 | 0 |
| 5 | 1 | 8 | 6 | 3 | 13 | 4 | 1 |
| *Do you consider that the tool has the ability to provide feedback?* | | | | | | | |
| 1 | 0 | 0 | 0 | 0 | 0 | 0 | 0 |
| 2 | 0 | 0 | 0 | 0 | 0 | 0 | 0 |
| 3 | 0 | 1 | 0 | 0 | 1 | 0 | 0 |
| 4 | 1 | 2 | 0 | 4 | 4 | 3 | 0 |
| 5 | 2 | 8 | 8 | 4 | 16 | 5 | 1 |
| *Did you feel the feedback provided was adequate?* | | | | | | | |
| 1 | 0 | 0 | 0 | 0 | 0 | 0 | 0 |
| 2 | 0 | 0 | 0 | 0 | 0 | 0 | 0 |
| 3 | 0 | 0 | 0 | 1 | 1 | 0 | 0 |
| 4 | 2 | 4 | 2 | 3 | 6 | 5 | 0 |
| 5 | 1 | 7 | 6 | 4 | 14 | 3 | 1 |
