# Supplementary material for: Use of a Low-Cost Portable 3D Virtual Reality Gesture-Mediated Simulator for Training and Learning Basic Psychomotor Skills in Minimally Invasive Surgery: Development and Content Validity Study
Source: J Med Internet Res. 2020 Jul 14;22(7):e17491. doi: 10.2196/17491 (PMC7388055; doi:10.2196/17491)
Supplement: Multimedia Appendix 3 [file jmir_v22i7e17491_app3.docx]

**Appendix 3. Results of the content validity survey.**

3.1 Content validity.

| **Content validity** | | | | | |
| --- | --- | --- | --- | --- | --- |
| **Capacity of training** | | | | | |
|  | **1** | **2** | **3** | **4** | **5** |
| Do you consider that the exercises allow the learning of hand-eye coordination? | 0 | 1 | 1 | 8 | 20 |
| Do you consider that the exercises allow the learning of depth perception? | 0 | 1 | 3 | 9 | 17 |
| Do you consider that the virtual environment allows the learning of basic psychomotor skills in laparoscopic surgery? | 0 | 0 | 1 | 8 | 21 |
| Do you consider that the virtual environment reflects the basic steps of any laparoscopic procedure? | 0 | 0 | 2 | 13 | 15 |
| Do you consider that the performance metrics provided (time, error, efficiency of movement, economy of diathermy) are adequate? | 0 | 0 | 5 | 11 | 13 |
| Do you consider that the prototype could become a solution for ubiquitous learning of basic psychomotor skills in laparoscopic surgery? | 0 | 0 | 0 | 11 | 19 |
| **Tasks** | | | | | |
|  | **1** | **2** | **3** | **4** | **5** |
| **Task 1. Grip and placement.** Does it reflect the grasping and retraction of a tissue into a given position? | 0 | 0 | 9 | 12 | 9 |
| **Task 2. Transfer and place.** Does it reflect the manipulation of a needle in an intracorporeal suture? | 1 | 2 | 4 | 13 | 10 |
| **Task 3. Cross.** Does it reflect exploration of the small intestine? | 1 | 2 | 5 | 12 | 10 |
| **Task 4. Removal and introduction.** Does it reflect the removal and introduction of the laparoscopic instruments? | 0 | 4 | 3 | 8 | 15 |
| **Task 5. Diathermy.** Does it reflect the cauterization of a blood vessel? | 0 | 0 | 3 | 8 | 19 |
| **Task 6. Manipulation and diathermy**. Does it reflect cauterization of the gallbladder bed? | 0 | 0 | 3 | 12 | 15 |

1. Strongly Disagree 2. Disagree 3. Neither agree nor disagree 4. Agree 5. Strongly agree

3.2 Content validity. Training capacity of the SIMISGEST-VR according to level of training.

|  |  |  |  |  |  |
| --- | --- | --- | --- | --- | --- |
| **Content validity** | **Total (n = 30)** | **Residents  (n = 8)** | **Practicing surgeon**  **(n = 21)** | **Other  (n = 1)** | **p** |
| Do you consider that the exercises allow the learning of hand-eye coordination? | 4.57 | 4.50 | 4.57 | 5.00 | 0.554 |
| Do you consider that the exercises allow the learning of depth perception? | 4.40 | 4.13 | 4.48 | 5.00 | 0.328 |
| Do you consider that the virtual environment allows the learning of basic psychomotor skills in laparoscopic surgery? | 4.67 | 4.38 | 4.76 | 5.00 | 0.266 |
| Do you consider that the virtual environment reflects the basic steps of any laparoscopic procedure? | 4.43 | 4.38 | 4.43 | 5.00 | 0.571 |
| Do you consider that the performance metrics provided (time, error, efficiency of movement, economy of diathermy) are adequate? | 4.28 | 4.00 | 4.35 | 5.00 | 0.375 |
| Do you consider that the prototype could become a solution for ubiquitous learning of basic psychomotor skills in laparoscopic surgery? | 4.63 | 4.38 | 4.71 | 5.00 | 0.187 |

3.3 Content validity. Capacity for training of the SIMISGEST-VR according to level of experience.

| **Content validity** | **Total**  **(n = 30)** | **Basic manipulation  (n = 3)** | **Basic operating level  (n = 11)** | **Intermediate operating level (n = 8)** | **Advanced operating level  (n = 8)** | **p** |
| --- | --- | --- | --- | --- | --- | --- |
| Do you consider that the exercises allow the learning of hand-eye coordination? | 4.57 | 4.33 | 4.73 | 4.63 | 4.38 | 0.656 |
| Do you consider that the exercises allow the learning of depth perception? | 4.40 | 4.67 | 4.36 | 4.38 | 4.38 | 0.940 |
| Do you consider that the virtual environment allows the learning of basic psychomotor skills in laparoscopic surgery? | 4.67 | 4.00 | 4.73 | 4.88 | 4.63 | 0.254 |
| Do you consider that the virtual environment reflects the basic steps of any laparoscopic procedure? | 4.43 | 4.33 | 4.45 | 4.75 | 4.13 | 0.231 |
| Do you consider that the performance metrics provided (time, error, efficiency of movement, economy of diathermy) are adequate? | 4.28 | 4.0 | 4.10 | 4.38 | 4.50 | 0.704 |
| Do you consider that the prototype could become a solution for ubiquitous learning of basic psychomotor skills in laparoscopic surgery? | 4.63 | 4.33 | 4.64 | 4.88 | 4.50 | 0.300 |

Basic manipulation of the camera and/or retraction with forceps

& Basic operating level (cholecystectomy, appendectomy)

+ Intermediate operating level (fundoplication)

^ Advanced operating level

3.4 Task according to level of training.

| **Task** | **Total  (n = 30)** | **Residents  (n = 8)** | **Practicing surgeon  (n = 21)** | **Other  (n = 1)** | **P** |
| --- | --- | --- | --- | --- | --- |
| Task 1 | 4.00 | 3.63 | 4.10 | 5.00 | 0.155 |
| Task 2 | 3.97 | 4.25 | 3.81 | 5.00 | 0.369 |
| Task 3 | 3.93 | 3.75 | 3.95 | 5.00 | 0.362 |
| Task 4 | 4.13 | 3.88 | 4.33 | 2.00 | 0.166 |
| Task 5 | 4.53 | 4.38 | 4.57 | 5.00 | 0.561 |
| Task 6 | 4.40 | 3.88 | 4.57 | 5.00 | **0.012** |

3.5 Task according to level of experience.

| **Task** | **Total  (n = 30)** | **Basic manipulation  (n = 3) *** | **Basic operating level  (n = 11) &** | **Intermediate level  (n = 8) +** | **Advanced level  (n = 8) ^** | ***p*** |
| --- | --- | --- | --- | --- | --- | --- |
| *Task 1* | 4.00 | 3.67 | 4.00 | 4.13 | 4.00 | 0.948 |
| *Task 2* | 3.97 | 4.33 | 4.18 | 4.13 | 3.38 | 0.959 |
| *Task 3* | 3.93 | 3.67 | 3.73 | 4.25 | 4.00 | 0.532 |
| *Task 4* | 4.13 | 3.00 | 4.09 | 4.75 | 4.00 | 0.104 |
| *Task 5* | 4.53 | 4.33 | 4.55 | 4.75 | 4.38 | 0.671 |
| *Task 6* | 4.40 | 4.00 | 4.18 | 4.88 | 4.38 | **0.04** |

* Basic manipulation of the camera and/or retraction with forceps

& Basic operating level (cholecystectomy, appendectomy)

+ Intermediate operating level (fundoplication)

^ Advanced operating level

3.6 Content validity vs levels of experience and training.

|  | **Level of experience** | | | | **Level of training** | | |
| --- | --- | --- | --- | --- | --- | --- | --- |
|  | **Basic manipulation  (n = 3)** | **Basic operating level  (n = 11)** | **Intermediate operating level  (n = 8)** | **Advanced operating level  (n = 8)** | **Practicing surgeon  (n = 21)** | **Resident  (n = 8)** | **Other  (n = 1)** |
| *Do you consider that the exercises allow the learning of hand-eye coordination?* | | | | | | | |
| 1 | 0 | 0 | 0 | 0 | 0 | 0 | 0 |
| 2 | 0 | 0 | 0 | 1 | 1 | 0 | 0 |
| 3 | 0 | 0 | 1 | 0 | 1 | 0 | 0 |
| 4 | 2 | 3 | 1 | 2 | 4 | 4 | 0 |
| 5 | 1 | 8 | 6 | 5 | 15 | 4 | 1 |
| *Do you consider that the exercises allow the learning of depth perception?* | | | | | | | |
| 1 | 0 | 0 | 0 | 0 | 0 | 0 | 0 |
| 2 | 0 | 0 | 1 | 0 | 1 | 0 | 0 |
| 3 | 0 | 2 | 0 | 1 | 1 | 2 | 0 |
| 4 | 1 | 3 | 2 | 3 | 6 | 3 | 0 |
| 5 | 2 | 6 | 5 | 4 | 13 | 3 | 1 |
| *Do you consider that the virtual environment allows the learning of the basic psychomotor skills in laparoscopic surgery?* | | | | | | | |
| 1 | 0 | 0 | 0 | 0 | 0 | 0 | 0 |
| 2 | 0 | 0 | 0 | 0 | 0 | 0 | 0 |
| 3 | 1 | 0 | 0 | 0 | 0 | 1 | 0 |
| 4 | 1 | 3 | 1 | 3 | 5 | 3 | 0 |
| 5 | 1 | 8 | 7 | 5 | 16 | 4 | 1 |
| *Do you consider that the virtual environment reflects the basic steps of any laparoscopic procedure?* | | | | | | | |
| 1 | 0 | 0 | 0 | 0 | 0 | 0 | 0 |
| 2 | 0 | 0 | 0 | 0 | 0 | 0 | 0 |
| 3 | 0 | 1 | 0 | 1 | 2 | 0 | 0 |
| 4 | 2 | 4 | 2 | 5 | 8 | 5 | 0 |
| 5 | 1 | 6 | 6 | 2 | 11 | 3 | 1 |
| *Do you consider that the metrics of performance provided (time, error, efficiency of movement, economy of diathermy) are adequate?* | | | | | | | |
| 1 | 0 | 0 | 0 | 0 | 0 | 0 | 0 |
| 2 | 0 | 0 | 0 | 0 | 0 | 0 | 0 |
| 3 | 1 | 3 | 1 | 0 | 2 | 3 | 0 |
| 4 | 1 | 3 | 3 | 4 | 9 | 2 | 0 |
| 5 | 1 | 4 | 4 | 4 | 9 | 3 | 1 |
| *Do you consider that the prototype could become a solution for ubiquitous learning of basic psychomotor skills in laparoscopic surgery?* | | | | | | | |
| 1 | 0 | 0 | 0 | 0 | 0 | 0 | 0 |
| 2 | 0 | 0 | 0 | 0 | 0 | 0 | 0 |
| 3 | 0 | 0 | 0 | 0 | 0 | 0 | 0 |
| 4 | 2 | 4 | 1 | 4 | 6 | 5 | 0 |
| 5 | 1 | 7 | 7 | 4 | 15 | 3 | 1 |

3.7 Task evaluation according to the level of experience and training

|  | **Level of experience** | | | | **Level of training** | | |
| --- | --- | --- | --- | --- | --- | --- | --- |
|  | **Basic manipulation  (n = 3)** | **Basic operating level  (n = 11)** | **Intermediate operating level  (n = 8)** | **Advanced operating level  (n = 8)** | **Practicing surgeon  (n = 21)** | **Resident  (n = 8)** | **Other  (n = 1)** |
| **Task 1** | | | | | | | |
| 1 | 0 | 0 | 0 | 0 | 0 | 0 | 0 |
| 2 | 0 | 0 | 0 | 0 | 0 | 0 | 0 |
| 3 | 2 | 3 | 2 | 2 | 5 | 4 | 0 |
| 4 | 0 | 5 | 3 | 4 | 9 | 3 | 0 |
| 5 | 1 | 3 | 3 | 2 | 7 | 1 | 1 |
| **Task 2** | | | | | | | |
| 1 | 0 | 0 | 0 | 1 | 1 | 0 | 0 |
| 2 | 0 | 0 | 0 | 2 | 2 | 0 | 0 |
| 3 | 0 | 1 | 2 | 1 | 4 | 0 | 0 |
| 4 | 2 | 7 | 3 | 1 | 7 | 6 | 0 |
| 5 | 1 | 3 | 3 | 3 | 7 | 2 | 1 |
| **Task 3** | | | | | | | |
| 1 | 0 | 1 | 0 | 0 | 1 | 0 | 0 |
| 2 | 1 | 0 | 0 | 1 | 1 | 1 | 0 |
| 3 | 0 | 2 | 2 | 1 | 4 | 1 | 0 |
| 4 | 1 | 6 | 2 | 3 | 7 | 5 | 0 |
| 5 | 1 | 2 | 4 | 3 | 8 | 1 | 1 |
| **Task 4** | | | | | | | |
| 1 | 0 | 0 | 0 | 0 | 0 | 0 | 0 |
| 2 | 2 | 1 |  | 1 | 1 | 2 | 1 |
| 3 | 0 | 1 | 1 | 1 | 3 | 0 | 0 |
| 4 | 0 | 5 | 0 | 3 | 5 | 3 | 0 |
| 5 | 1 | 4 | 7 | 3 | 12 | 3 | 0 |
| **Task 5** | | | | | | | |
| 1 | 0 | 0 | 0 | 0 | 0 | 0 | 0 |
| 2 | 0 | 0 | 0 | 0 | 0 | 0 | 0 |
| 3 | 0 | 2 | 0 | 1 | 2 | 1 | 0 |
| 4 | 2 | 1 | 2 | 3 | 5 | 3 | 0 |
| 5 | 1 | 8 | 6 | 4 | 14 | 4 | 1 |
| **Task 6** | | | | | | | |
| 1 | 0 | 0 | 0 | 0 | 0 | 0 | 0 |
| 2 | 0 | 0 | 0 | 0 | 0 | 0 | 0 |
| 3 | 0 | 2 | 0 | 1 | 2 | 1 | 0 |
| 4 | 3 | 5 | 1 | 3 | 5 | 7 | 0 |
| 5 | 0 | 4 | 7 | 4 | 14 | 0 | 1 |
